# The Influence of Trait Compulsivity and Impulsivity on Addictive and Compulsive Behaviors During COVID-19

**DOI:** 10.3389/fpsyt.2021.634583

**Published:** 2021-02-23

**Authors:** Lucy Albertella, Kristian Rotaru, Erynn Christensen, Amelia Lowe, Mary-Ellen Brierley, Karyn Richardson, Samuel R. Chamberlain, Rico S. C. Lee, Edouard Kayayan, Jon E. Grant, Sam Schluter-Hughes, Campbell Ince, Leonardo F. Fontenelle, Rebecca Segrave, Murat Yücel

**Affiliations:** ^1^BrainPark, Turner Institute for Brain and Mental Health, Monash University, Clayton, VIC, Australia; ^2^Monash Business School, Monash University, Caulfield, VIC, Australia; ^3^Department of Psychiatry, University of Southampton, Southampton, United Kingdom; ^4^Department of Psychiatry and Behavioral Neuroscience, University of Chicago, Chicago, IL, United States; ^5^Obsessive, Compulsive, and Anxiety Spectrum Research Program, Institute of Psychiatry, Federal University of Rio de Janeiro (UFRJ), D'Or Institute for Research and Education (IDOR), Rio de Janeiro, Brazil; ^6^D'Or Institute for Research and Education, Rio de Janeiro, Brazil

**Keywords:** compulsivity, impulsivity, addiction, OCD, COVID-19

## Abstract

**Background:** The COVID-19 pandemic has resulted in high levels of psychological distress worldwide, with experts expressing concern that this could result in corresponding increases in addictive behaviors as individuals seek to cope with their distress. Further, some individuals may be at greater risk than others for developing problematic addictive behaviors during times of high stress, such as individuals with high trait impulsivity and compulsivity. Despite the potential of such knowledge to inform early detection of risk, no study to date has examined the influence of trait impulsivity and compulsivity on addictive behaviors during COVID-19. Toward this aim, the current study examined the association between impulsive and compulsive traits and problematic addictive and compulsive behaviors during the first COVID-19 lockdown in Australia.

**Methods:** Eight hundred seventy-eight adults completed a cross-sectional online survey during the first lockdown, between late May to June 2020. Participants completed scales for addictive and compulsive behaviors for the period prior to and during lockdown for problematic eating, pornography, internet use, gambling, drinking, and obsessive-compulsive behaviors. Negative binomial regressions examined the associations between impulsivity, compulsivity, and their interaction with problematic behaviors during lockdown, controlling for age, gender, sample, psychological distress, exposure to COVID-related stressors, and pre-COVID problems.

**Results:** Greater trait compulsivity was associated with more problematic obsessive-compulsive behaviors (*p* < 0.001) and less problematic drinking (*p* = 0.038) during lockdown. Further, trait compulsivity interacted with trait impulsivity in relation to problematic eating behaviors (*p* = 0.014) such that greater trait compulsivity was associated with more problems among individuals with low impulsivity only (*p* = 0.030). Finally, psychological distress and/or exposure to COVID-related stressors were associated with greater problems across all addictive and compulsive behaviors, as was severity of pre-COVID problems.

**Discussion:** Trait compulsivity was associated with addictive and compulsive behaviors in different ways. Further, the finding that stress-related variables (psychological distress and COVID-related stressors) were associated with greater problems across all lockdown behaviors supports the idea that stress may facilitate, or otherwise be associated with, problematic behaviors. These findings highlight the need for interventions that enhance resilience to stress, which in turn may reduce risk for addictive and compulsive disorders.

## Introduction

Stress is a well-known risk factor across addictive and compulsive behaviors (1, 2). This knowledge has led to the general expectation that such behaviors will increase during the COVID-19 pandemic (3–6), considered a stressful time worldwide due to health and financial concerns, lockdown-related social isolation, and life disruption. While studies suggest that some addictive and compulsive behaviors may have increased during COVID-19, including problematic internet use (7), drinking (8), and obsessive-compulsive behaviors (9), this has not been the case across the board. Particularly, reports of gambling-related harm suggest a decrease during lockdown (10, 11), and there have been mixed findings for obsessive-compulsive behaviors [e.g., (12)]. An emerging body of research suggests that lockdown-related changes in addictive and compulsive behaviors may be predicted by, or otherwise related to, behavior-specific factors, such as motives [e.g., (13)] and pre-existing severity (6, 10, 14). However, individual characteristics also play a role [e.g., (15)]. This pattern of findings is not unique to COVID-19; there is a wealth of past research showing that while stressful life events generally increase risk for addictive and compulsive behaviors (16–19), the extent to which they do is influenced by individual differences (20–22). As such, COVID-19 provides an invaluable context within which to better understand (and thereby address) individual-level risk factors for psychopathology.

It is generally accepted that, at least under non-pandemic circumstances, trait impulsivity is associated with risk *across* the spectrum of addictive and compulsive disorders (23–31). Briefly, impulsivity refers to the tendency to act without thinking, especially when the consequences of such action are inappropriate to the situation (32, 33). There is a large body of evidence showing that greater trait impulsivity is associated with more problematic addictive and compulsive behaviors, including for alcohol use, gambling, internet use, binge eating, pornography, as well as obsessive-compulsive behaviors (24, 30, 34–40). Another risk factor for addictive and compulsive behaviors is compulsivity, that is, the tendency to engage in repetitive, habitual behaviors that are difficult to control or interfere with current goals (27, 41–46). Indeed, higher levels of trait compulsivity have been found to be associated with addictive and compulsive behaviors, including problematic alcohol use, internet use, binge eating, gambling, and obsessive-compulsive behaviors (35, 37, 46, 47). Further, research suggests that impulsivity and compulsivity may interact such that individuals with high levels on both compulsive and impulsive traits are at greatest risk of problematic impulsive-compulsive behaviors (23, 29, 35). For instance, individuals characterized by high impulsivity and high compulsivity have been shown to have more severe obsessive-compulsive symptoms (29) and problematic eating (48). Similarly, this interaction is seen at the cognitive level, with higher levels of both impulsive and compulsive cognitive traits being associated with more problematic alcohol use and obsessive-compulsive behaviors (35).

Arguably, this risk profile (high impulsivity, high compulsivity) might contribute to more problematic addictive and compulsive behaviors during lockdown. For instance, while individuals with high impulsivity and low compulsivity might engage in impulsive behaviors during lockdown, they would not engage in the same impulsive behavior routinely. On the other hand, individuals with high compulsivity and low impulsivity might engage in certain behaviors routinely during lockdown but might be able to inhibit these newly adopted routine behaviors should they become maladaptive. However, when these traits are combined, an individual might engage in routine coping behaviors (due to compulsive tendencies) and have difficulty inhibiting these behaviors if they become maladaptive (due to the impaired response inhibition that characterizes impulsivity). Thus, individuals with high compulsivity and high impulsivity may be at greater risk of developing persistent, maladaptive coping behaviors during the current pandemic. This risk may further increase with time, as impulsive behaviors become coping strategies (through reinforcement) and routine behaviors become habits. Intervening early in the course of impulsive-compulsive behaviors, before behaviors become entrenched, is critical to curtailing progression to addictive and compulsive disorders (44).

Early detection of risk for impulsive-compulsive disorders may be especially important during the current pandemic as problematic behaviors may become entrenched more quickly under times of high stress. Specifically, stress may facilitate progression toward problematic compulsive behaviors by promoting a shift toward habit learning and/or otherwise supporting the maladaptive expression of learned behaviors (44, 49–54). Through facilitating these mechanisms, stress may effectively shorten the window of time that a behavioral pattern is malleable. Thus, early detection of risk during COVID-19 (a stressful period for many) is critical to enabling timely access to interventions, before addictive and compulsive behaviors become harder to modify. The current study therefore aimed to examine the potential of trait compulsivity and impulsivity as risk markers for problematic addictive and compulsive behaviors during the first lockdown of COVID-19. Specifically, this study examined the associations between trait compulsivity, impulsivity, and their interaction on problematic internet use, drinking, eating, pornography use, gambling, and obsessive-compulsive behaviors during COVID-19. Obsessive-compulsive behaviors were examined alongside addictive behaviors in line with transdiagnostic models of compulsive behaviors (42, 44, 55), as well as the recent conceptualization of OCD as a behavioral addiction (56). In line with the idea that impulsive and compulsive traits may pre-dispose individuals to developing problematic behaviors, especially during times of high stress, we hypothesized that impulsivity and compulsivity would interact in relation to problematic behaviors during lockdown. Specifically, we hypothesized that individuals with high compulsivity *and* high impulsivity would report the greatest increases in addictive and compulsive behaviors during lockdown.

## Method

### Participants

Participants included in the study were 992 adults (18 years and above). The current analyses exclude participants who did not complete all the general study measures (trait impulsivity and compulsivity, COVID events, and psychological distress), which were 114 in total. Thus, the resulting study sample includes 878 participants. Participants were recruited through two methods: (1) general advertisements on Facebook, twitter, and other social media platforms, and reimbursement was entry into a draw to win one of 50 $100 JB HiFi vouchers, and (2) Prolific online participant recruitment platform targeting individuals residing in Australia, and reimbursement was £7.50 per hour. The current study includes 214 community participants and 664 prolific participants.

All study procedures were carried out in accordance with the Declaration of Helsinki. The Monash University Human Research Ethics Committee ethically reviewed and approved the study.

### Measures

Demographic information such as age and gender was collected, and participants completed the following questionnaires:

**Short UPPS-P Impulsivity Scale** [S-UPPS-P; (57)]: This is a 20-item scale that measures impulsivity traits with five subscales: Negative Urgency, the tendency toward impulsive action when experiencing strong negative emotions (e.g., “When I am upset, I often act without thinking”); Positive Urgency, the tendency toward impulsive action when experiencing strong positive emotions; Lack of Perseverance; Lack of Premeditation; and Sensation Seeking. For each item, participants selected whether the extent to which they agreed or disagreed with statements describing ways in which people act and think (generally, i.e., no timeframe was specified). Response options were “strongly disagree,” “disagree somewhat,” “agree somewhat,” or “strongly agree,” scored as 1–4, respectively (or 4–1 for reverse items). The present study used total S-UPPS-P score as the measure of interest.

**The Cambridge-Chicago Compulsivity Trait Scale** [CHI-T; (47)]. This is a 15-item scale covering broad aspects of compulsivity including the need for completion or perfection, being stuck in a habit, reward-seeking, desire for high standards, and avoidance of situations that are hard to control. For each item, participants selected whether the statement applied to them (generally, i.e., no timeframe was specified) by selecting “strongly disagree,” “disagree,” “agree,” or “strongly agree,” scored as 0–3, respectively. The measure of interest was the total score.

**COVID-related events**: An 8-item checklist of COVID-related events was used to gauge exposure to stressors from the start of the pandemic. These eight items were taken from a measure of potentially stressful COVID-related events [COROTRAS; (58, 59)]. Specifically, these items asked about worsening of financial situation; reduced time in paid employment; being diagnosed with COVID-19; having a family member or significant other diagnosed with COVID-19; having experienced a cough or fever during the pandemic; being kept away from home (in another state or country) because of COVID-19; having family member or significant other share space with a suspected or confirmed case of COVID-19 or being in a position where they are exposed to lots of people; and having to work or be exposed against your wishes to any activity associated with a high risk of contracting COVID-19. The measure was in the form of a checklist (with a score of 1 given for each event experienced) the total score was used in the present study (i.e., total number of events experienced).

**K10** (60): This is a 10-item scale designed to measure past month psychological distress. Each item is rated on a 5-point scale as follows: None of the time (1); A little of the time (2); Some of the time (3); Most of the time (4); or All of the time (5). The measure of interest was the total score. We adjusted for psychological distress given research showing that it is associated with increases in addictive behaviors during COVID-19 (61) as well as its elevation during COVID-19 (62, 63). The total score was used in the present study.

#### Problematic Behavior Scales

**Modified Yale Food Addiction Scale 2.0** [mYFAS2.0; (64)]: This scale is a 13-item scale designed to measure addiction-like eating behaviors in accordance with the DSM5 diagnostic criteria for addictive disorders, with additional items asking about distress and interference as a result of the eating behaviors. All participants completed the mYFAS 2.0. The scale was modified to cover a month timeframe and response options were modified as follows: Never (1); 1–3 times/month (2); 1–3 times/week; (3); 4+ times/week (4). Further, each scale item was asked in relation to both (a) the month prior to the onset of the first COVID-19 restrictions and (b) the past month, during COVID-19 restrictions. The current study used total scores for each timeframe (pre-COVID and lockdown) as the measures of interest.

**Young's Internet Addiction Test, Short Version** [IAT; (65)]: This is a 12-item version of Young's IAT developed to measure Problematic Usage of the Internet. Only participants who reported excessive use of the internet in the past 3 months were asked to complete the IAT. Each scale item was asked in relation to both (a) the month prior to the onset of the first COVID-19 restrictions and (b) the past month, during COVID-19 restrictions. Item response options were as follows: Never (0); Rarely (1); Sometimes; (2); Often (3); and Very often (4). The current study used total scores for each timeframe (pre-COVID and lockdown) as the measures of interest.

**Short Version of the Problematic Pornography Consumption Scale** [PPCS-6; (40)]: This is a 6-item scale designed to measure problematic pornography use. Only participants who reported watching pornography in the past 3 months were asked to complete the PPCS-6. Each scale item was asked in relation to both (a) the month prior to the onset of the first COVID-19 restrictions and (b) the past month, during COVID-19 restrictions. Item response options were as follows: Never (1); Sometimes; (2); Often (3); and Very often (4). The current study used total scores for each timeframe (pre-COVID and lockdown) as the measures of interest.

**Problem Gambling Severity Index** [PGSI; derived from the 31-item Canadian Problem Gambling Index, (66)]. This is a 9-item measure of gambling harm severity. Only participants who reported gambling in the past 3 months were asked to complete the PGSI. Each scale item was asked in relation to both (a) the month prior to the onset of the first COVID-19 restrictions and (b) the past month, during COVID-19 restrictions. Item response options were as follows: Never (0); Sometimes; (1); Almost always (2); and Always (3). The current study used total scores for each timeframe (pre-COVID and lockdown) as the measures of interest.

**Alcohol Use Disorders Identification Test** [AUDIT; (67)]. The AUDIT is a 10-item self-report measure that assesses hazardous/risky alcohol consumption. Only participants who reported drinking in the past 3 months were asked to complete the AUDIT. Each scale item was asked in relation to both (a) the month prior to the onset of the first COVID-19 restrictions and (b) the past month, during COVID-19 restrictions. Response options were modified to suit the 1-month timeframe needed for the current study. For questions 1, response options were: Never (0); Once a month (1); 2–4 times/month (2); 2–3 times/week (3); 4+ times/week. For questions 3–8, response options were: Never (0); Monthly (1); Weekly (2); Daily or almost daily (3). For questions 9 and 10, participants were asked to answer yes (2) or no (0) in relation to the timeframe in question. The current study used total scores for each timeframe (pre-COVID and lockdown) as the measures of interest.

**Obsessive-Compulsive Inventory Revised** [OCI-R; (68)]. This is an 18-item scale enquiring about OC-related experiences. All participants were asked to complete the OCI-R. Each scale item was asked in relation to both (a) the month prior to the onset of the first COVID-19 restrictions and (b) the past month, during COVID-19 restrictions. For each scale item the individual rated how distressed or bothered they had been by this over the specified timeframe, with response options as follows: Not at all (0), A little (1), Moderately (2), A lot (3), or Extremely (4). The current study used total scores for each timeframe (pre-COVID and lockdown) as the measures of interest.

### Statistical Analyses

The data were examined for outliers (based on Z scores >3.29), which were then winsorized. Descriptive statistics compared pre-COVID to lockdown problematic behaviors using Wilcoxon Signed Ranks Test (Table 1), and examined correlations across compulsivity, impulsivity, and all problematic behaviors during lockdown (Table 2). Six negative binomial regressions examined whether trait impulsivity (S-UPPS-P score), trait compulsivity (CHIT score), and their interaction were associated with each of the following problematic behaviors during lockdown; eating, internet use, pornography use, drinking, gambling, and obsessive-compulsive behaviors. Compulsivity scores and impulsivity scores were mean-centered according to the respective outcome group, and interaction terms calculated accordingly. All regression models adjusted for corresponding pre-COVID problematic behavior score, age, gender, sample, COVID-related events, and psychological distress (K10).

**Table 1:** (A) Sample descriptives (*N* = 878) and (B) Pre-COVID and lockdown problematic behavior scale scores.

**(A)**		**Overall sample**	
Gender	% female	53%	
Age	M	32.0	
	SD	12.50	
Impulsivity	M	42.7	
	SD	7.40	
Compulsivity	M	26.8	
	SD	5.48	
Distress	Md/M Range/SD	20/21.8	
		10–50/78.8	
COVID stressors	Md/M Range/SD	2/1.6	
		0–5/1.7	
**(B)**		**Pre-COVID**	**Lockdown**
Eating (*n* = 878)	Md/M Range/SD	15/17.0 13–36/5.5	15/17.4 13–38/6.1
Pornography (*n* = 438)	Md/M Range/SD	8/9.0 6–19/3.0	8/9.2 6–20/3.2
Gambling (*n* = 150)	Md/M Range/SD	1/2.9 0–19/4.7	0/2.3 0–14/3.7
Internet (*n* = 375)	Md/M Range/SD	15/16.2 0–42/8.0	18/19.5 0–48/9.2
Alcohol (*n* = 599)	Md/M Range/SD	4/4.6 0–18/3.8	3/4.5 0–18/3.8
OCS (*n* = 878)	Md/M Range/SD	1/4.4 0–27/6.6	3/6.0 0–32/7.7

*NB. Impulsivity, trait impulsivity (measured using the S-UPPS-P); Compulsivity, trait compulsivity (measured using the CHI-T); Distress, psychological distress (measured using the K10); Eating, problematic eating (measured using the mYFAS 2.0, modified for 1-month timeframe); Pornography, problematic pornography use (measured using the PPCS); Gambling, problematic gambling (measured using the PGSI, modified for 1-month timeframe); Internet, problematic internet use (measured using the IAT); Alcohol, Problematic alcohol use (measured using the AUDIT, modified for 1-month timeframe); OCS, obsessive-compulsive symptoms (measured using the OCI-R)*.

**Table 2:** Spearman's correlation across impulsivity, compulsivity, and problematic behaviors during lockdown.

		**1**	**2**	**3**	**4**	**5**	**6**	**7**	**8**
		**Imp**	**Comp**	**Eating**	**Pornography**	**Gambling**	**Internet**	**Alcohol**	**OCS**
1	rs	1.000							
	*p*	–							
	*N*	878							
2	rs	**0.181**	1.000						
	*p*	**<0.001**	–						
	*N*	**878**	878						
3	rs	**0.195**	**0.221**	1.000					
	*p*	**<0.001**	**<0.001**	–					
	*N*	**878**	**878**	878					
4	rs	**0.238**	**0.245**	**0.253**	1.000				
	*p*	**<0.001**	**<0.001**	**<0.001**	–				
	*N*	**438**	**438**	**438**	438				
5	rs	**0.329**	**0.216**	**0.370**	**0.406**	1.000			
	*p*	**<0.001**	**0.008**	**<0.001**	**<0.001**	–			
	*N*	**150**	**150**	**150**	**93**	150			
6	rs	**0.208**	**0.201**	**0.305**	**0.349**	**0.340**	1.000		
	*p*	**<0.001**	**<0.001**	**<0.001**	**<0.001**	**0.006**	–		
	*N*	**375**	**375**	**375**	**210**	**65**	375		
7	rs	**0.173**	0.022	**0.127**	**0.112**	**0.311**	0.075	1.000	
	*p*	**<0.001**	0.583	**0.002**	**0.043**	**0.001**	0.233	–	
	*N*	**599**	599	**599**	**329**	**117**	251	599	
8	rs	**0.205**	**0.471**	**0.414**	**0.356**	**0.348**	**0.475**	**0.093**	1.000
	*p*	**<0.001**	**<0.001**	**<0.001**	**<0.001**	**<0.001**	**<0.001**	**0.022**	–
	*N*	**878**	**878**	**878**	**438**	**150**	**375**	**599**	878

*NB. Imp, trait impulsivity (measured using the S-UPPS-P); Comp, trait compulsivity (measured using the CHI-T); Eating, problematic eating (measured using the mYFAS 2.0, modified for 1-month timeframe); Pornography, problematic pornography use (measured using the PPCS); Gambling, problematic gambling (measured using the PGSI, 1-month timeframe); Internet, problematic internet use (measured using the IAT); Alcohol, Problematic alcohol use (measured using the AUDIT, modified for 1-month timeframe); OCS, obsessive-compulsive symptoms (measured using the OCI-R). Bolded font signifies p <0.05*.

Significant and trend-level interactions were followed up by dividing the sample into high and low trait impulsivity groups (by median split, according to corresponding outcome group) and running a negative binomial regression with trait compulsivity as the predictor, lockdown score of behavior in question as the dependent variable, and adjusting for the pre-COVID scale score.

Further, to provide an illustration of significant interactions, we graphed change scores (calculated as lockdown minus pre-COVID score) by high and low impulsivity and compulsivity groups (median split). This is shown in the Supplementary Figure 1. Finally, to support interpretation of study findings, pre-COVID behaviors were analyzed to examine their relationship with trait impulsivity and compulsivity. These analyses are also presented in the Supplementary Materials.

## Results

Participants were 878 adults (466 females; age *M* = 32.0 years, *SD* = 12.5, range 18–84). Prolific participants were younger than community participants [mean diff. = 2.5, *t*_(876)_ = 2.5, *p* = 0.012]. The community sample had relatively more females (71 vs. 47%) than the prolific sample, *X*^2^ = 36.6, *p* < 0.001. The community sample also reported higher lockdown obsessive-compulsive symptoms scores than the prolific sample, *Z* = −2.5, *p* = 0.012. No other differences were found between the two samples.

As shown in Table 1, problematic internet use, *Z* = 12.0, *p* < 0.001, *d*_*Cohen*_ = 0.98, pornography use, *Z* = 3.5, *p* < 0.001, *d*_*Cohen*_ = 0.24, eating, *Z* = 5.5, *p* < 0.001, *d*_*Cohen*_ = 0.27, and obsessive-compulsive symptoms, *Z* = 15.0, *p* < 0.001, *d*_*Cohen*_ = 0.77, increased from pre-COVID to lockdown. In contrast, problematic gambling score decreased from pre-COVID to lockdown, *Z* = −2.6, *p* = 0.011, *d*_*Cohen*_ = 0.30. No differences were found for problematic drinking. As shown in Table 2, trait compulsivity and impulsivity were significantly correlated with all lockdown behaviors, except for problematic drinking, which did not show a significant correlation with trait compulsivity.

### Problematic Eating During Lockdown

Results of the regression on lockdown problematic eating are shown in Table 3. Female gender was associated with increased problematic eating during lockdown (*Wald X*^2^ = 9.7, *p* = 0.002), as was greater psychological distress (*Wald X*^2^ = 27.0, *p* < 0.001), and higher pre-COVID problematic eating score (*Wald X*^2^ = 1,343.4, *p* < 0.001). The interaction between trait compulsivity and impulsivity was also significant (*Wald X*^2^ = 6.3, *p* = 0.014). Follow-up of this interaction found that while the association between compulsivity scores and lockdown eating was significant for the low impulsivity group (*Wald X*^2^ = 4.7, *p* = 0.030, *n* = 423), it was not significant in the high impulsivity group (*Wald X*^2^ = 0.61, *p* = 0.434, n = 455). Supplementary Figure 1 shows change scores (calculated as lockdown minus pre-COVID score) by high and low impulsivity and compulsivity groups (median split), to aid interpretation of the above interaction.

**Table 3:** Regression results.

	**B**	**SE**	**LCI**	**UCI**	**Wald *X*^**2**^**	***p***
Sample	0.008	0.0113	−0.014	0.030	0.462	0.497
**Gender**	**0.030**	**0.0097**	**0.011**	**0.049**	**9.358**	**0.002**
Age	7.84E-5	0.0004	−0.001	0.001	0.032	0.858
COVID stressors	0.005	0.0044	−0.004	0.013	1.065	0.302
**Psych. Distress**	**0.004**	**0.0008**	**0.002**	**0.005**	**26.985**	**<0.001**
Comp	3.60E-4	0.0009	−0.001	0.002	0.144	0.704
Imp	−0.001	0.0007	−0.002	0.001	0.974	0.324
**Imp** **×** **Comp**	**−2.47E-4**	**0.0001**	**4.44E-4**	**−5.04E-5**	**6.061**	**0.014**
**Pre-COVID score**	**0.044**	**0.0012**	**0.042**	**0.046**	**1343.364**	**<0.001**

*DV: problematic eating behaviors during lockdown (N = 878). Bolded font signifies p < 0.05*.

### Problematic Pornography Use During Lockdown

Results of the regression on lockdown problematic pornography use are shown in Table 4. Female gender was associated with lower lockdown problematic pornography use (*Wald X*^2^ = 15.5, *p* < 0.001). Younger age (*Wald X*^2^ = 5.2, *p* = 0.023), a higher number of COVID events (*Wald X*^2^ = 5.4, *p* = 0.020), and greater pre-COVID problematic pornography use (*Wald X*^2^ = 674.3, *p* < 0.001) were associated with higher lockdown problematic pornography use. Finally, there was a trend-level interaction (*Wald X*^2^ = 3.2, *p* = 0.076), which follow-up analyses revealed was driven by a trend-level association between compulsivity and lockdown pornography use in the low impulsivity group (*Wald X*^2^ = 3.2, *p* = 0.072, *n* = 224) which was not seen in the high impulsivity group (*Wald X*^2^ = 0.48, *p* = 0.488, *n* = 214).

**Table 4:** Regression results.

	**B**	**SE**	**LCI**	**UCI**	**Wald *X*^**2**^**	***p***
Sample	−0.030	0.0227	−0.075	0.014	1.788	0.181
**Gender**	**−0.065**	**0.0164**	**−0.097**	**−0.032**	**15.523**	**<0.001**
**Age**	**−0.001**	**0.0006**	**−0.003**	**−2.08E-4**	**5.203**	**0.023**
**COVID stressors**	**0.015**	**0.0064**	**0.002**	**0.028**	**5.404**	**0.020**
Psych. Distress	0.002	0.0010	−0.001	0.004	2.091	0.148
Comp	3.91E-4	0.0013	−0.002	0.003	0.088	0.766
Imp	0.001	0.0010	−0.001	0.003	1.419	0.234
Imp × Comp	−2.36E-4	0.0001	−4.97E-4	2.45E-5	3.153	0.076
**Pre-COVID score**	**0.087**	**0.0033**	**0.080**	**0.093**	**674.297**	**<0.001**

*DV: problematic pornography use during lockdown (N = 438). Bolded font signifies p <0.05*.

### Problematic Gambling During Lockdown

Results of the regression on lockdown problematic gambling scores are shown in Table 5. Younger age (*Wald X*^2^ = 13.3, *p* < 0.001), greater psychological distress (*Wald X*^2^ = 6.0, *p* = 0.014), and greater pre-COVID problematic gambling (*Wald X*^2^ = 56.4, *p* < 0.001) were associated with more problematic gambling during lockdown.

**Table 5:** Regression results.

	**B**	**SE**	**LCI**	**UCI**	**Wald *X*^**2**^**	***p***
Sample	0.184	0.3859	−0.573	0.940	0.226	0.634
Gender	−0.188	0.2606	−0.699	0.323	0.519	0.471
**Age**	**−0.040**	**0.0108**	**−0.061**	**−0.018**	**13.342**	**<0.001**
COVID stressors	0.092	0.0835	−0.072	0.255	1.208	0.272
**Psych. Distress**	**0.027**	**0.0111**	**0.005**	**0.049**	**6.021**	**0.014**
Comp	−0.030	0.0252	−0.079	0.019	1.412	0.235
Imp	−0.006	0.0199	−0.044	0.033	0.078	0.781
Imp x Comp	0.001	0.0029	−0.005	0.006	0.035	0.852
**Pre-COVID score**	**0.223**	**0.0297**	**0.165**	**0.282**	**56.445**	**<0.001**

*DV: problematic gambling behaviors during lockdown (N = 150). Bolded font signifies p < 0.05*.

### Problematic Internet Use During Lockdown

Results of the regression on lockdown problematic internet use are shown in Table 6. Younger age (*Wald X*^2^ = 8.9, *p* = 0.003), community sample status (*Wald X*^2^ = 10.3, *p* = 0.001), greater K10 (*Wald X*^2^ = 11.1, *p* = 0.001), and greater pre-COVID problematic internet use (*Wald X*^2^ = 204.3, *p* < 0.001), were associated with more problematic internet use during lockdown.

**Table 6:** Regression results.

	**B**	**SE**	**LCI**	**UCI**	**Wald *X*^**2**^**	***p***
**Sample**	**−0.149**	**0.0466**	**−0.241**	**−0.058**	**10.254**	**0.001**
Gender	−0.006	0.0332	−0.071	0.059	0.037	0.848
**Age**	**−0.005**	**0.0018**	**−0.009**	**−0.002**	**8.907**	**0.003**
COVID stressors	0.002	0.0181	−0.033	0.038	0.017	0.897
**Psych. Distress**	**0.009**	**0.0026**	**0.004**	**0.014**	**11.057**	**0.001**
Comp	−0.002	0.0027	−0.008	0.003	0.705	0.401
Imp	−0.002	0.0031	−0.008	0.004	0.586	0.444
Imp × Comp	−1.70E-4	0.0004	−0.001	0.001	0.221	0.639
**Pre-COVID score**	**0.047**	**0.0033**	**0.040**	**0.053**	**204.309**	**<0.001**

*DV: problematic internet use during lockdown (N = 375). Bolded font signifies p < 0.05*.

### Problematic Drinking During Lockdown

Results of the regression on lockdown problematic drinking scores are shown in Table 7. Older age (*Wald X*^2^ = 6.6, *p* = 0.010), greater COVID-related events (*Wald X*^2^ = 9.3, *p* = 0.002), lower trait compulsivity (*Wald X*^2^ = 4.3, *p* = 0.038), and greater pre-COVID drinking problems (*Wald X*^2^ = 316.1, *p* < 0.001) were associated with more problematic drinking during lockdown.

**Table 7:** Regression results.

	**B**	**SE**	**LCI**	**UCI**	**Wald *X*^**2**^**	***p***
Sample	−0.013	0.0551	−0.121	0.095	0.055	0.814
Gender	−0.020	0.0470	−0.112	0.072	0.177	0.674
**Age**	**0.005**	**0.0018**	**0.001**	**0.008**	**6.600**	**0.010**
**COVID stressors**	**0.059**	**0.0194**	**0.021**	**0.097**	**9.348**	**0.002**
Psych. Distress	0.002	0.0032	−0.004	0.008	0.412	0.521
**Comp**	**−0.009**	**0.0043**	**−0.018**	**−4.88E-4**	**4.294**	**0.038**
Imp	−0.001	0.0037	−0.008	0.007	0.021	0.884
Imp × Comp	0.001	0.0006	−2.71E-4	0.002	2.315	0.128
**Pre-COVID score**	**0.132**	**0.0074**	**0.117**	**0.146**	**316.089**	**<0.001**

*DV: problematic alcohol use during lockdown (N = 599). Bolded font signifies p < 0.05*.

### Problematic Obsessive-Compulsive Behaviors During Lockdown

Results of the regression on problematic obsessive-compulsive behaviors during lockdown are shown in Table 8. Younger age (*Wald X*^2^ = 4.5, *p* = 0.033), greater COVID-related events (*Wald X*^2^ =9.2, *p* = 0.002), greater psychological distress (*Wald X*^2^ = 38.6, *p* < 0.001), greater trait compulsivity (*Wald X*^2^ = 38.8, *p* < 0.001), and greater pre-COVD obsessive-compulsive behaviors (*Wald X*^2^ = 319.9, *p* < 0.001) were associated with more problematic obsessive-compulsive behaviors during lockdown.

**Table 8:** Regression results.

	**B**	**SE**	**LCI**	**UCI**	**Wald *X*^**2**^**	***p***
Sample	−0.096	0.0791	−0.251	0.059	1.472	0.225
Gender	0.028	0.0679	−0.105	0.162	0.176	0.675
**Age**	**−0.006**	**0.0028**	**−0.011**	**−4.85E-4**	**4.562**	**0.033**
**COVID stressors**	**0.083**	**0.0273**	**0.029**	**0.137**	**9.208**	**0.002**
**Psych. Distress**	**0.028**	**0.0045**	**0.019**	**0.037**	**38.643**	**<** **0.001**
**Comp**	**0.044**	**0.0071**	**0.030**	**0.058**	**38.803**	**<0.001**
Imp	0.002	0.0053	−0.008	0.013	0.186	0.667
Imp × Comp	−0.001	0.0009	−0.003	0.001	1.532	0.216
**Pre-COVID score**	**0.106**	**0.0059**	**0.094**	**0.118**	**319.865**	**<0.001**

*DV: problematic obsessive-compulsive behaviors during lockdown (N = 878). Bolded font signifies p < 0.05*.

### Supplementary Analyses on Pre-COVID Problematic Behaviors

Higher trait impulsivity and/or compulsivity, or their interaction were significantly associated with all pre-COVID problematic behaviors. Please see Supplementary Materials for details.

## Discussion

The current study examined whether two transdiagnostic risk factors, trait impulsivity and compulsivity, and their interaction, were associated with problematic addictive and compulsive behaviors during lockdown. First, the current study found that participants reported increased problematic behaviors during lockdown, compared to pre-COVID levels, except for alcohol use and gambling. In fact, participants reported reduced gambling during lockdown. However, with the exception of reported changes (from pre-COVID to lockdown) in obsessive-compulsive symptoms and internet use, which were large in effect size, reported changes in problematic behaviors were small in effect size. Second, trait impulsivity and compulsivity were significantly correlated with all lockdown problematic behaviors (except compulsivity with alcohol use). These correlations were small to medium in effect size and generally in line with past research in non-clinical populations (35, 36, 38). However, these relationships changed considerably once examined within regression models, which controlled for pre-COVID levels of problematic behaviors. These analyses found that greater trait compulsivity was associated with greater lockdown obsessive-compulsive behaviors, as well as lower levels of lockdown problematic drinking. Further, trait compulsivity interacted with impulsivity in relation to problematic eating and (at trend level) pornography use. Follow-up of these interactions found that greater trait compulsivity was associated with greater problematic eating and (at trend-level) pornography use during lockdown among individuals with *low* trait impulsivity only. It must be noted however that the effect sizes of these interactions are very small, as may be seen from Tables 3, 4 (interaction term *B*s). Psychological distress and/or exposure to COVID-related stressors were associated with greater problems across all addictive and compulsive lockdown behaviors as were pre-COVID levels of the behavior in question.

The finding that greater trait compulsivity was associated with more problematic obsessive-compulsive behaviors during lockdown, after adjusting for psychological distress, COVID-related stressors, and pre-COVID obsessive-compulsive behaviors highlights its role as a key risk marker for OCD. While the nature of its role in driving risk has yet to be identified, the current findings suggest that these traits, or what they reflect, interact with environmental factors to promote the expression of compulsive symptoms. Critically, while greater compulsivity was associated with obsessive-compulsive behaviors during lockdown, it was not associated with *pre-COVID* obsessive-compulsive behaviors (expect through interaction with impulsivity; see Supplementary Table 6 for details). Notably, trait compulsivity is associated with family history of obsessive-compulsive and addictive behaviors (46). Thus, these traits may reflect a genetic predisposition toward compulsivity that is influenced by environmental factors (69). As the nature of COVID-19 stressors directly support OCD symptomatology (e.g., contamination concerns), this pre-disposition (which is reflected in trait compulsivity) might then be expected to be associated with greater obsessive-compulsive symptoms during lockdown, more so than with other compulsive and addictive behaviors during lockdown. Finally, this finding adds to the growing literature supporting the CHI-T scale as a measure that is sensitive to OCD-related risk in the general population (28, 46, 47), and may be especially useful to detect at-risk individuals who might benefit from early intervention during the pandemic to minimize progression and entrenchment of problematic behaviors.

Higher trait compulsivity was also associated with more problematic eating behaviors during lockdown, albeit among individuals with low impulsivity only. Among individuals with high impulsivity, trait compulsivity was not associated with problematic lockdown eating behaviors. This pattern of findings may reflect the high impulsivity group having higher levels of pre-existing problematic eating (see Supplementary Table 1), which was itself associated with greater problematic eating during lockdown. In contrast, the lower levels of baseline eating problems among individuals with low impulsivity may have allowed for other influences on lockdown behavior to be revealed, such as trait compulsivity. This pattern of findings was also seen at trend-level for problematic pornography use and may be interpreted similarly. Finally, greater trait compulsivity was associated with lower problematic alcohol use during lockdown. This finding may be best understood in the context of lockdown-related closures of public venues where drinking was common prior to COVID-19. For individuals who drank at these venues regularly, these places provided a wide range of cues (people, situation, etc.) and routines that supported drinking. Individuals high on trait compulsivity are habit- and routine-oriented (47, 70), and strongly influenced by cues (46). Thus, with the closure of public drinking venues, compulsive individuals who drank there lost the cues and routines that previously promoted their drinking. According to this account, without such routines and cues to promote drinking, compulsive individuals may drink less during lockdown than previously, at least, until new drinking habits and routines set in.

The finding that higher psychological distress was associated with greater problematic behaviors during lockdown is in line with emerging findings across addictive and compulsive behaviors (8, 10, 61, 71), as well as a large body of literature suggesting that stress facilitates habit-driven behavior and/or otherwise promotes the maladaptive expression of learned behaviors (44, 49–53). Problematic obsessive-compulsive behaviors were associated with both COVID-related events and psychological distress, in line with a recent study using a COVID events checklist (from which the current items were taken) in relation to obsessive-compulsive and related disorders (59). These findings may be explained in various ways. For instance, for people with obsessive-compulsive tendencies, COVID-related events might be more salient, which may in turn increase reporting of them. Supporting this interpretation, pre-COVID obsessive-compulsive behaviors were the only pre-COVID problematic behavior (of all addictive and compulsive behaviors) associated with exposure to COVID-19 events (see Supplementary Table 6). Further, as several COVID-related events involve potential harm to others and/or contamination, exposure to these events may further promote compulsive behaviors through triggering obsession-related concerns.

In line with other COVID-19 studies, greater pre-COVID levels of problematic behaviors predicted greater problematic behaviors during lockdown across all problematic behaviors. This provides important context for interpreting the current findings in relation to trait impulsivity and compulsivity and their role in driving risk during the current pandemic. That is, while their relationship with addictive and compulsive behaviors is evident from past research (24, 25, 46), as well as current findings (see Supplementary Tables), they may have limited influence on behavior during the current pandemic at this early stage, at least, over and above stress-related influences and pre-COVID levels of the behavior in question. It is likely that the influence of trait impulsivity and compulsivity will become clearer over time, as patterns of behavior become established and differences emerge in relation to how people adapt their behaviors as problems arise. In any case, the current findings highlight the need to better understand the different roles that individual risk factors might play during life as usual vs. during COVID-19, and how these traits might interact with environmental factors to influence disorder-specific expressions.

The current study has several limitations, such as its cross-sectional design, which limits the ability to draw conclusions about the direction of the findings. For instance, while we interpreted the association between compulsivity and problematic eating as indicating that compulsivity increases risk for problematic eating (in those with low impulsivity), an alternative explanation might be that engaging in excessive, unhealthy eating may result in cognitive impairments that in turn drive inflexible, compulsive behaviors (72, 73). Longitudinal research is needed to better understand the direction of the relationship between the trait impulsivity and compulsivity and how they are related to problematic behaviors over the course of this pandemic. Other limitations include the self-reporting of problematic behaviors, including comparisons of behaviors at different timepoints, which is subject to bias and random error. However, previous studies have found self-reported addictive and obsessive-compulsive behavior measures to be generally valid and reliable (74, 75). Also, the current study did not control for important confounding variables such as current mental health diagnosis, trauma, psychiatric medication, illicit drug use, or IQ. Such variables have been shown to be associated with addictive and compulsive behaviors (76–80) as well as impulsivity and/or compulsivity (81–83). Future studies are needed to confirm the present findings taking these confounding variables into account. Finally, participants in this study were recruited through social media and may therefore not be representative of individuals in the general population.

A clear implication of the current findings is the need for interventions that increase resilience to stress to protect against its effects on addictive and compulsive behaviors. Such interventions may include promoting adaptive coping skills and/or healthy lifestyle patterns. For instance, engaging in exercise has been shown to reduce stress levels acutely (84) and regular exercise has been shown to increase resilience to stress generally (85) and has been linked to greater resilience during COVID-19 (63, 86, 87). Further, maintaining a healthy diet (88) and having strong social support (89) have also been linked to increased resilience to stress generally, as well as during COVID-19 (61, 87, 90). Through enhancing resilience to stress, lifestyle interventions and the use of adaptive coping strategies may in turn reduce the risk for addictive and compulsive behaviors during the COVID-19 pandemic.

In conclusion, the current study found that the influence of trait impulsivity and compulsivity on addictive and compulsive behaviors during lockdown differed according to the behavior in question. These behavior-specific findings suggest that traits may interact with situational factors to influence whether pre-existing behaviors continue, increase, or decrease during major life disruptions. In contrast, stress-related variables, i.e., psychological distress and/or exposure to COVID-related stressors, were associated with greater problems across all addictive and compulsive behaviors. The current study adds to the growing literature supporting the need for interventions that enhance resilience to stress during the current pandemic, which in turn could reduce risk for addictive and compulsive disorders.

## Contribution to the Field

The COVID-19 pandemic has resulted in high levels of psychological distress worldwide, with experts expressing concern that this could result in corresponding increases in addictive behaviors as individuals seek to cope with their distress. People with high levels of impulsive and compulsive traits may be especially prone to developing problematic coping behaviors during COVID-19. Not only do these traits heighten risk generally, but their influence on risk may be accelerated during times of stress. Thus, early detection of risk is critical as the timeframe for early intervention may be shortened by stress. The current study thus examined the potential of impulsive and compulsive traits to serve as risk markers for addictive and compulsive behaviors during COVID-19. The findings suggest that while impulsive-compulsive traits were associated with all problematic pre-COVID behaviors examined, their influence was limited to a few problematic behaviors during COVID-19. In contrast, stress-related variables were associated with all problematic behaviors during COVID-19, as was severity of pre-COVID problems. These findings suggest that the influence of impulsive and compulsive traits on addictive behaviors during COVID-19 might be largely indirect, mediated through pre-COVID problems. Further, these findings also highlight the impact of stress-related factors across addictive and compulsive behaviors and the need for interventions aimed at enhancing resilience to stress, which in turn may reduce risk for addictive and compulsive disorders.

## Data Availability Statement

The raw data supporting the conclusions of this article will be made available by the authors, without undue reservation.

## Ethics Statement

The studies involving human participants were reviewed and approved by Monash Human Research Ethics Committee. The patients/participants provided their consent to participate in this study.

## Author Contributions

LA wrote first draft of this manuscript. LA, MY, KRo, and RS designed the major components of the study. LA, KRo, EC, M-EB, AL, and KRi contributed critically to data collection for this study. All authors contributed to revising subsequent versions of the paper. All authors contributed to the selection of study measures.

## Conflict of Interest

The authors declare that the research was conducted in the absence of any commercial or financial relationships that could be construed as a potential conflict of interest.
