# Supplementary material for: The Influence of Trait Compulsivity and Impulsivity on Addictive and Compulsive Behaviors During COVID-19
Source: Front Psychiatry. 2021 Feb 23;12:634583. doi: 10.3389/fpsyt.2021.634583 (PMC7940765; doi:10.3389/fpsyt.2021.634583)
Supplement: Supplementary file 1 [file Data_Sheet_1.PDF]

SUPPLEMENTARY MATERIALS

TO ACCOMPANY

The influence of trait compulsivity and impulsivity on addictive and compulsive behaviours during COVID-19

Lucy Albertella[1], Kristian Rotaru[1,2], Erynn Christensen[1], Amelia Lowe[1], Mary-Ellen Brierley[1], Karyn Richardson[1], Samuel R. Chamberlain[3], Rico S.C Lee[1], Edouard Kayayan[1], Jon E. Grant[4], Sam Schluter-Hughes[1], Campbell Ince[1], Leonardo F. Fontenelle[1,5,6], Rebecca Segrave[1]*, and Murat Yücel[1]*

***shared senior authors**

[1]BrainPark, Turner Institute for Brain and Mental health, Monash University, Victoria, Australia
[2]Monash Business School, Monash University, Australia
[3]Department of Psychiatry, University of Southampton, Southampton, UK
[4]Department of Psychiatry & Behavioral Neuroscience, University of Chicago, USA
[5]Obsessive, Compulsive, and Anxiety Spectrum Research Program. Institute of Psychiatry, Federal University of Rio de Janeiro (UFRJ) & D'Or Institute for Research and Education (IDOR), Rio de Janeiro, Brazil
[6]D'Or Institute for Research and Education, Rio de Janeiro, Brazil

This document contains the following information:

1. Analyses of pre-COVID problematic behaviours (Tables 1-6)

2. Graph representing problematic eating change score for low and high impulsivity and compulsivity groups (Figure 1)

Analyses of pre-COVID problematic behaviours

Six negative binomial regressions examined whether trait impulsivity (S-UPPS-P score), trait compulsivity (CHIT score), or their interaction were associated with various problematic behaviours in the month *prior* to the first COVID-19 lockdown in Australia. Problematic behaviours included: eating (Table 1), internet use (Table 2), pornography use (Table 3), drinking (Table 4), gambling (Table 5), and obsessive-compulsive behaviours (Table 6). CHI-T scores and S-UPPS-P scores were mean-centred according to the respective outcome group, and interaction terms calculated accordingly. All regression models adjusted for age, gender, sample, COVID-related events, and psychological distress (K10).

Table 1. Regression results. DV: Pre-COVID problematic eating behaviours (N = 878).

	B	SE	LCI	UCI	Wald X^2	*p*
Sample	-.018	.0229	-.063	.027	.620	.431
Gender	.035	.0189	-.002	.072	3.430	.064
Age	.001	.0008	-.001	.002	.676	.411
COVID stressors	-.001	.0083	-.017	.015	.012	.913
**Psych. Distress**	**.012**	**.0013**	**.010**	**.015**	**82.627**	**<.001**
**Comp**	**.005**	**.0019**	**.001**	**.009**	**6.454**	**.011**
**Imp**	**.006**	**.0013**	**.004**	**.009**	**24.194**	**<.001**
Imp x Comp	.000	.0002	-.001	.000	.425	.514

NB. 'Comp' = trait compulsivity (measured using the CHI-T); 'Imp' = trait impulsivity (measured using the S-UPPS-P).

Table 2. Regression results. DV: Pre-COVID problematic internet use (N = 375).

	B	SE	LCI	UCI	Wald X^2	*p*
Sample	.037	.8989	-1.725	1.799	.002	.967
**Gender**	**-1.731**	**.7535**	**-3.208**	**-.254**	**5.276**	**.022**
**Age**	**-.099**	**.0342**	**-.166**	**-.031**	**8.285**	**.004**
COVID stressors	-.117	.3023	-.709	.476	.149	.700
**Psych. Distress**	**.295**	**.0504**	**.197**	**.394**	**34.310**	**<.001**
Comp	.110	.0729	-.033	.253	2.285	.131
**Imp**	**.133**	**.0541**	**.027**	**.239**	**6.036**	**.014**
Imp x Comp	-.004	.0077	-.019	.011	.252	.616

NB. 'Comp' = trait compulsivity (measured using the CHI-T); 'Imp' = trait impulsivity (measured using the S-UPPS-P).

Table 3. Regression results. DV: Pre-COVID problematic pornography use (N = 438).

	B	SE	LCI	UCI	Wald X^2	*p*
Sample	.033	.0362	-.038	.104	.835	.361
**Gender**	**-.216**	**.0281**	**-.271**	**-.160**	**58.900**	**<.001**
Age	-.001	.0015	-.004	.002	.454	.500
COVID stressors	.006	.0111	-.015	.028	.335	.563
**Psych. Distress**	**.006**	**.0019**	**.002**	**.010**	**9.945**	**.002**
**Comp**	**.009**	**.0026**	**.004**	**.014**	**11.269**	**.001**
**Imp**	**.007**	**.0020**	**.003**	**.011**	**13.551**	**<.001**
Imp x Comp	7.335E-5	.0003	-.001	.001	.057	.361

NB. 'Comp' = trait compulsivity (measured using the CHI-T); 'Imp' = trait impulsivity (measured using the S-UPPS-P).

Table 4. Regression results. DV: Pre-COVID problematic alcohol use (N = 599).

	B	SE	LCI	UCI	Wald X^2	*p*
Sample	-.035	.0731	-.178	.108	.230	.632
**Gender**	**-.198**	**.0620**	**-.319**	**-.076**	**10.170**	**.001**
**Age**	**.010**	**.0025**	**.005**	**.015**	**15.319**	**<.001**
COVID stressors	.038	.0232	-.008	.083	2.667	.102
**Psych. Distress**	**.014**	**.0040**	**.006**	**.022**	**12.251**	**<.001**
Comp	.007	.0066	-.006	.020	1.024	.311
**Imp**	**.028**	**.0041**	**.020**	**.036**	**47.607**	**<.001**
Imp x Comp	.000	.0007	-.001	.002	.055	.815

NB. 'Comp' = trait compulsivity (measured using the CHI-T); 'Imp' = trait impulsivity (measured using the S-UPPS-P).

Table 5. Regression results. DV: Pre-COVID problematic gambling behaviours (N = 150).

	B	SE	LCI	UCI	Wald X^2	*p*
**Sample**	**1.004**	**.4370**	**.147**	**1.860**	**5.276**	**.022**
**Gender**	**-.631**	**.2741**	**-1.169**	**-.094**	**5.307**	**.021**
Age	.013	.0118	-.010	.036	1.179	.277
COVID stressors	-.016	.0835	-.180	.147	.038	.846
Psych. Distress	.021	.0128	-.004	.046	2.634	.105
**Comp**	**.059**	**.0280**	**.004**	**.114**	**4.431**	**.035**
**Imp**	**.073**	**.0161**	**.042**	**.105**	**20.855**	**.000**
Imp x Comp	-.002	.0035	-.009	.005	.286	.593

NB. 'Comp' = trait compulsivity (measured using the CHI-T); 'Imp' = trait impulsivity (measured using the S-UPPS-P).

Table 6. Regression results. DV: Pre-COVID problematic obsessive-compulsive behaviours (N = 878).

	B	SE	LCI	UCI	Wald X^2	*p*
Sample	-.342	.1091	-.556	-.128	9.823	.002
Gender	-.130	.1000	-.326	.066	1.688	.194
**Age**	-.008	.0043	-.016	.001	3.078	.079
**COVID stressors**	**.063**	**.0062**	**.051**	**.075**	**102.481**	**<.001**
**Psych. Distress**	**.085**	**.0366**	**.013**	**.156**	**5.328**	**.021**
Comp	.086	.0102	.066	.106	70.822	<.001
Imp	.023	.0083	.006	.039	7.476	.006
**Imp x Comp**	**-.005**	**.0011**	**-.007**	**-.003**	**18.911**	**<.001**

NB. 'Comp' = trait compulsivity (measured using the CHI-T); 'Imp' = trait impulsivity (measured using the S-UPPS-P).

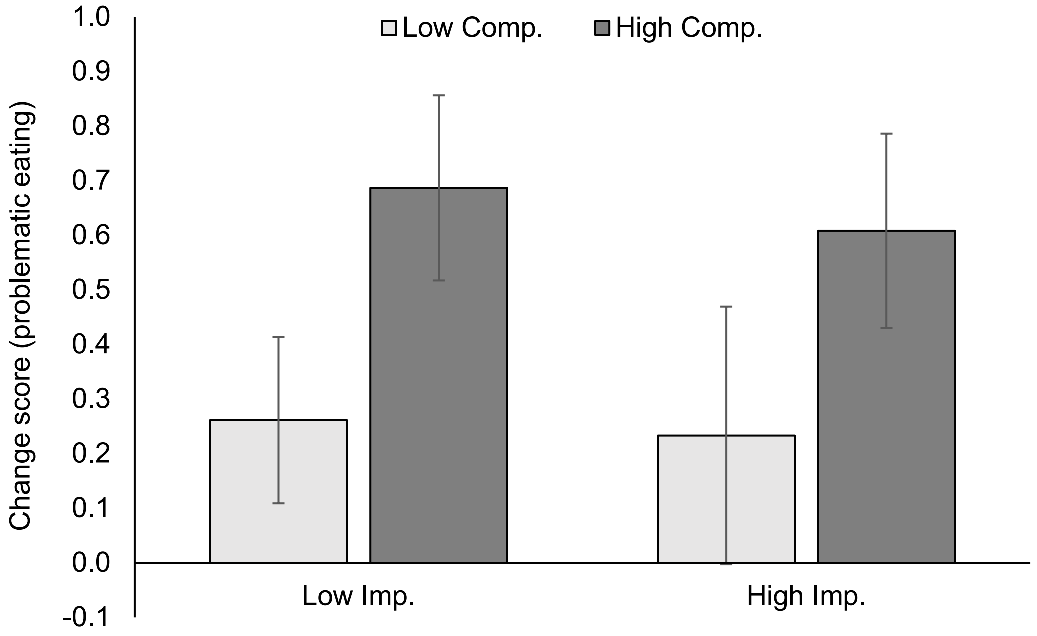

Figure 1. Graph representing change score* in problematic eating for low and high impulsivity and compulsivity groups

** Change score was calculated as lockdown score minus pre-COVID score*
